# Quantitative Structure–Retention Relationships with Non-Linear Programming for Prediction of Chromatographic Elution Order

**DOI:** 10.3390/ijms20143443

**Published:** 2019-07-12

**Authors:** J. Jay Liu, Alham Alipuly, Tomasz Bączek, Ming Wah Wong, Petar Žuvela

**Affiliations:** 1 Department of Chemical Engineering, Pukyong National University, Busan 48-513, Korea; 2 Department of Pharmaceutical Chemistry, Medical University of Gdańsk, Al. Gen. Hallera 107, 80-416 Gdańsk, Poland; 3 Department of Chemistry, National University of Singapore, 3 Science Drive 3, Singapore 117543, Singapore

**Keywords:** quantitative structure-retention relationships (QSRR), chromatography, reversed phase-liquid chromatography (RP-LC), elution order prediction, non-linear programming (NLP)

## Abstract

In this work, we employed a non-linear programming (NLP) approach via quantitative structure–retention relationships (QSRRs) modelling for prediction of elution order in reversed phase-liquid chromatography. With our rapid and efficient approach, error in prediction of retention time is sacrificed in favor of decreasing the error in elution order. Two case studies were evaluated: (i) analysis of 62 organic molecules on the Supelcosil LC-18 column; and (ii) analysis of 98 synthetic peptides on seven reversed phase-liquid chromatography (RP-LC) columns with varied gradients and column temperatures. On average across all the columns, all the chromatographic conditions and all the case studies, percentage root mean square error (%RMSE) of retention time exhibited a relative increase of 29.13%, while the %RMSE of elution order a relative decrease of 37.29%. Therefore, sacrificing %RMSE(*t*_R_) led to a considerable increase in the elution order predictive ability of the QSRR models across all the case studies. Results of our preliminary study show that the real value of the developed NLP-based method lies in its ability to easily obtain better-performing QSRR models that can accurately predict both retention time and elution order, even for complex mixtures, such as proteomics and metabolomics mixtures.

## 1. Introduction

Quantitative structure–retention relationships (QSRRs) [1,2] modelling has become a de-facto standard for the prediction of retention time in reversed-phase liquid chromatography analysis, which accounts for > 90% of separations in modern laboratories [3]. QSRRs and retention prediction in general have numerous applications. From identification of the most informative structural molecular descriptors with respect to retention mechanisms, prediction of retention for new analytes, up to comparison of different chromatographic columns and determination of physical properties (lipophilicity, dissociation constants, relative bioactivities).

Elution order in reversed phase-liquid chromatography (RP-LC) is typically governed by polarity of the mobile phase, whereby the more hydrophobic the analytes are, the longer it takes for them to elute (decreasing polarity) [4]. For simple analytical mixtures (e.g., < 10 analytes), it is straightforward to predict their elution order based on hydrophobicity (expressed as log*P*) which can be determined either experimentally or in silico. However, nowadays, chromatographers face analytical mixtures with ever-increasing complexity (e.g., proteomics, wastewater, pharmaceutical mixtures) which can lead to chromatograms comprised of thousands of close and even overlapping peaks. In this case, a retention time prediction model with a low error does not guarantee the same for elution order.

There are only a few studies in literature dealing with the problem of elution order prediction in RP-LC including our previous work where we presented a multi-objective-optimization (MOO)-based method [4,5,6,7]. For instance, Vorslova et al. [5] present a study for prediction of retention times of phenylisothiocyanate derivatives of 25 natural amino acids using gradient RP-LC. The two-parameter solvatic sorption QSRR model with three physicochemical constants was used for prediction of the retention times. Namely, the electrostatic interaction energy of analytes with water, partial molar volume of analytes in water, surface tension and dielectric permittivity values for both the mobile and stationary phases, and a constant which includes the phase ratio and other characteristics of both stationary and mobile phases. The authors have reported average deviations between predicted and experimental retention time values of < 6%, while the predicted elution order mostly corresponded to the experimental ones, with some larger deviations for retention times > 15 minutes, with several unresolved (simulated) peaks.

Shinoda et al. [6] have used artificial neural networks (ANNs) to model the retention times of peptides with up to 50 amino acid residues. The authors report a good model for 834 peptides (with the determination coefficient, *R*^2^ of 0.928). The QSRR model is further applied to a dataset of 121,273 peptides resulting from LysC-digestion of the *Escherichia coli* proteome, however without experimental validation. The developed ANN-based QSRR model has also been used to predict elution order for improvement of peptide identifications in reversed phase-liquid chromatography / tandem mass spectrometry (RP-LC-MS/MS) workflows. Elution order of peptides was predicted with an error of < 11%. The method itself was based on prediction of anteroposterior relations of each peptide pair. However, the details of the methodology are not very well described. On the other hand, Bach et al. [7] presented a complex machine learning-based methodology for prediction of elution order in metabolomics based on rank support vector machines and dynamic programming.

The developed QSRR models were based on molecular fingerprints of two molecules as input and elution order as output. The authors postulate that elution order is far more conserved across different columns and instruments than retention time seemingly overcoming the main limitation of QSRR. However, the results of the elution order predictions are quite sensitive to the composition and number of training samples, while the developed method itself is computationally intensive [7].

In our previous work [4], we have presented an MOO-based elution order prediction method using genetic algorithms (GA) [8,9] for optimization employing two QSRR models with a priori selected molecular descriptors related to the RP-LC retention mechanism. Although the presented results were quite promising, showing “positive” trends (i.e., considerable decrease in elution order errors, with an increase of retention time errors), GA required considerable computing times of several minutes, whereas the execution of the multiple linear regression–non-linear programming (MLR-NLP) is nearly instantaneous. On top of that, the interior-point algorithm used to solve the NLP formulation of elution order is much less complex than GA.

**Figure 1:**
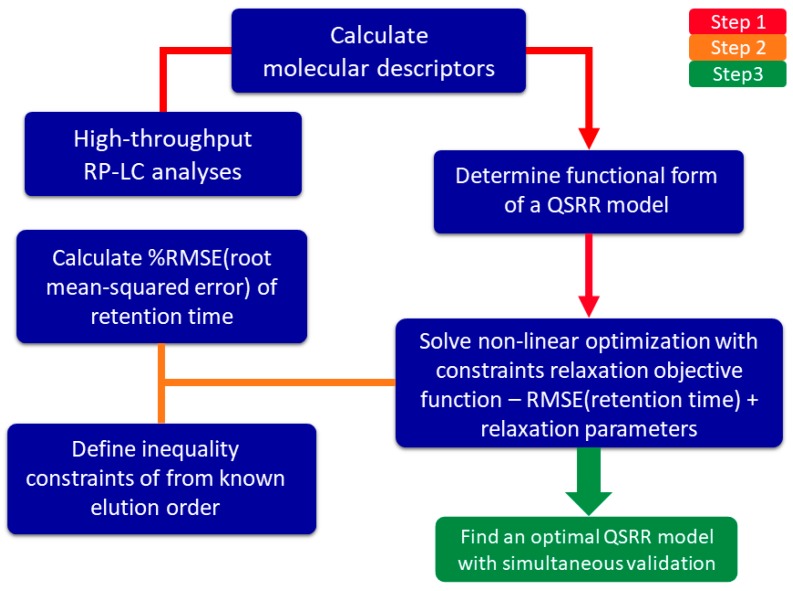
Schematic depiction of the non-linear programming (NLP)-based elution order prediction methodology. Abbreviations (in order of appearance): RP-LC—reversed-phase liquid chromatography, RMSE—root mean square error, QSRR—quantitative structure-retention relationships.

In this work, we have defined elution order prediction as an NLP problem (Figure 1) with relaxed constraints; considerably faster compared to the MOO-based method. The developed NLP-based method is directly implemented within the QSRR modelling process and was used for prediction of elution order of two (more simple) analytical mixtures: (i) analysis of 62 organic molecules on the Supelcosil LC-18 column; and (ii) analysis of 98 synthetic peptides on seven RP-LC columns with varied gradients and column temperatures. Results are compared to the QSRR models built using only multiple linear regression (MLR) [10] termed control models.

## 2. Results and Discussion

In this work, an NLP-based formulation directly implemented within the QSRR modelling process has been derived for prediction of chromatographic elution order in RP-LC. The method was applied to two case studies with rather simple separations on seven columns in varied chromatographic conditions. 

Two QSRR models were evaluated: one for RP-LC separation of organic compounds, and the other for the RP-LC separation of peptides. MLR was used to construct “control” models, while an NLP formulation was formed to solve the problem of elution order prediction. The two were compared in terms of performance and with the paired *t*-test.

**Figure 2:**
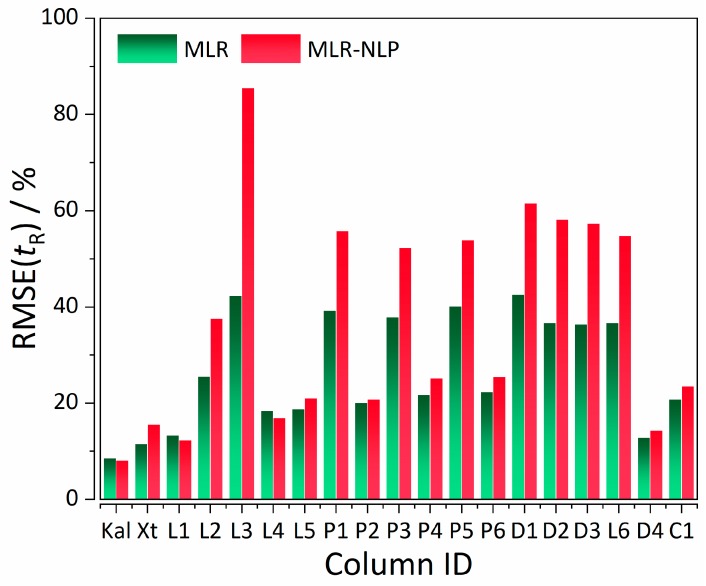
QSRR model performance expressed in terms of %RMSE(*t*_R_) for MLR (control) and MLR-NLP models. Legend: Kal—Supelcosil LC18, *t*_G_ = 10 min, *T* = 35 °C (case study 1); Xt—Xterra, *t*_G_ = 20 min, *T* = 40 °C; L1—Licrospher, *t*_G_ = 20 min, *T* = 40 °C; L2—*t*_G_ = 60 min, *T* = 40 °C; L3—*t*_G_ = 120 min, *T* = 40 °C; L4—*t*_G_ = 20 min, *T* = 60 °C; L5—*t*_G_ = 20 min, *T* = 80 °C; L6—Licrospher CN, *t*_G_ = 20 min, *T* = 40 °C; P1—PRP, *t*_G_ = 20 min, *T* = 40 °C; P2—*t*_G_ = 60 min, *T* = 40 °C; P3—*t*_G_ = 20 min, *T* = 60 °C; P4—*t*_G_ = 60 min, *T* = 60 °C; P5—*t*_G_ = 20 min, *T* = 80 °C; P6—*t*_G_ = 60 min, *T* = 80 °C; D1—Discovery RP-Amide C-16, *t*_G_ = 20 min, *T* = 40 °C; D2—*t*_G_ = 20 min, *T* = 60 °C; D3—*t*_G_ = 20 min, *T* = 80 °C; D4—Discovery HS F5-3, *t*_G_ = 20 min, *T* = 40 °C; C1—Chromolith, *t*_G_ = 20 min, *T* = 40 °C (case study 2). Abbreviations: QSRR—quantitative structure-retention relationships; %RMSE(*t*_R_)—percentage root mean square error of retention time.

**Figure 3:**
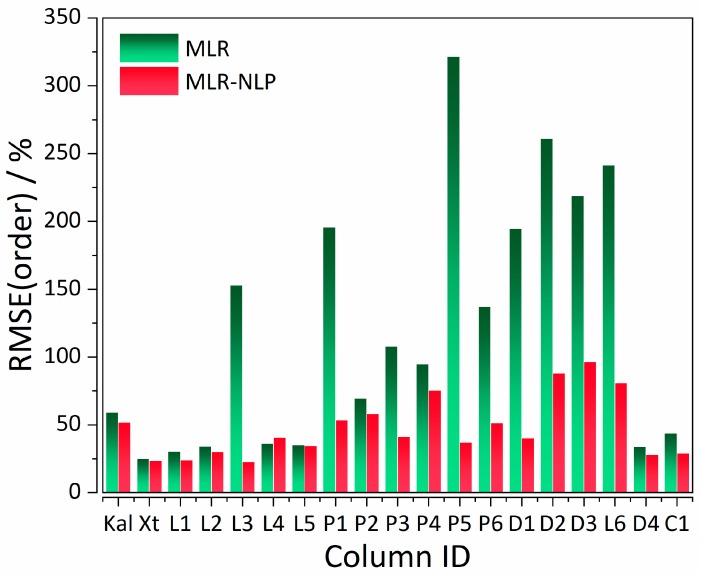
Distribution of %RMSE (order) values of MLR (control) and MLR-NLP models. The legend for the X-axis is analogous to the one in Figure 2.

As it can be observed from Figure 2 and Figure 3, most of the columns follow a "positive" trend; with the increase of retention time %RMSE, %RMSE of elution order considerably decreases, with a few exceptions (Kaliszan 1, Licrospher 1, and Licrospher 4).

**Figure 4:**
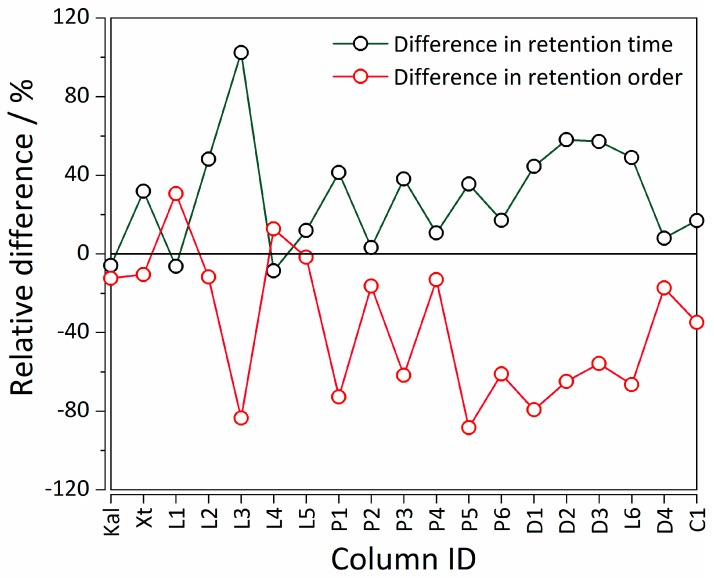
Relative difference in retention time and elution order %RMSE values between MLR and MLR-NLP models. The legend for the X-axis is analogous to the one in Figure 2. Abbreviations: %RMSE—percentage root mean square error, MLR—multiple linear regression, MLR-NLP—MLR–non-linear programming.

Statistical significance of the differences between the QSRR model performances for all the columns between the two methods (MLR and MLR-NLP) has been tested with a paired *t*-test. Table 1 summarizes the *t*-test results and it was shown that the two approaches exhibit statistically significant differences (*p* < 0.05). The relative differences between %RMSE in retention time and elution order are evident from Figure 4, with deviations from the “positive” trend for three chromatographic columns, in which the NLP-based method surprisingly exhibited better performance than the MLR control models in terms of %RMSE(*t*_R_).

In fact, one of the chromatographic columns, Supelcosil LC, has exhibited a decrease in both %RMSE(*t*_R_) and %RMSE(order). These deviations can be explained with the non-linearity between the parameters calculated from the molecular structure of the analytes and their retention times. Thereby, for the columns in question, our formulation has led to a better QSRR model. The MLR model itself is fully linear, whereas our NLP-based formulation introduces a degree of non-linearity due to its multinomial quadratic form (see Section 3.4.).

**Figure 5:**
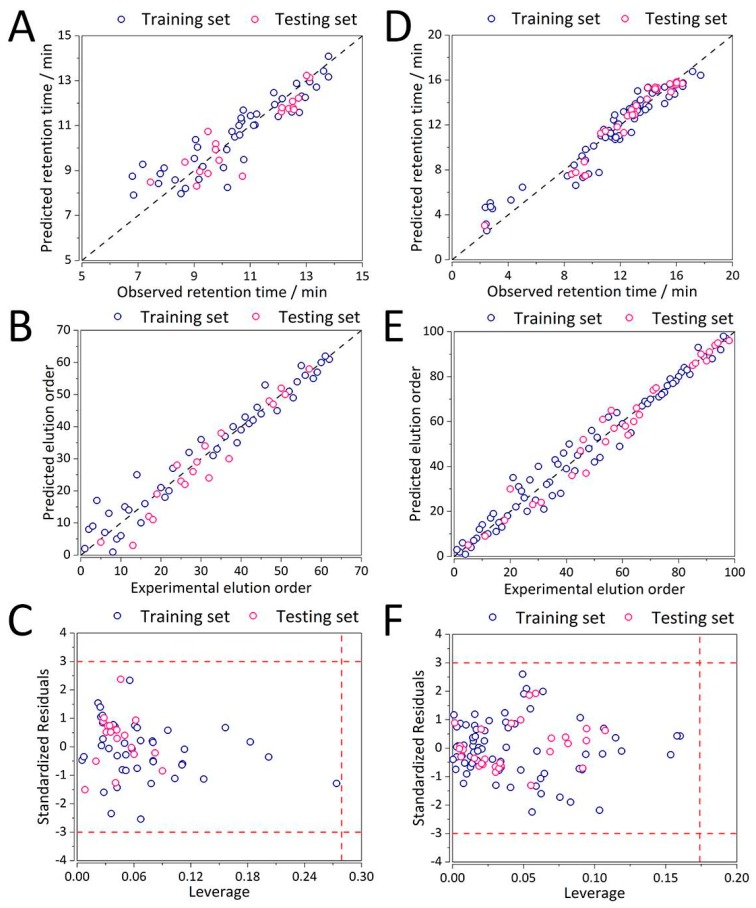
Performance of the MLR-NLP method for prediction of (**A**) retention time, (**B**) elution order, and (**C**) applicability domain for case study 1 (separation of organic molecules using Supelcosil LC, *t*_G_ = 10 min, *T* = 35 °C), (**D**) prediction of retention time, (**E**) elution order, and (**F**) applicability domain for case study 2 (separation of synthetic peptides on Xterra, *t*_G_ = 20 min, *T* = 40 °C). Abbreviations: *t*_G_—gradient retention time, *T*—temperature.

Detailed results for both case studies and all the columns/chromatographic conditions are summarized in Table 2, while the performance plots for all the columns are available in the Supporting Information (Figures S1–S10). Out of the evaluated chromatographic columns / conditions, two exemplary QSRR models from both case studies were detailed here (Supelcosil LC with *t*_G_ = 10 min, *T* = 35 °C and Xterra with *t*_G_ = 20 min, *T* = 40 °C). Both the NLP-based QSRR models have exhibited low %RMSE(*t*_R_) of 8.07% and 15.17% (Table 2), with the former decreasing, and the latter increasing in comparison to the control MLR models. This can also be observed from the predictive ability plots in Figure 5A,D. The respective %RMSE(order) were 51.77% and 22.4% (Table 2). In both cases the larger errors in elution order seem to originate from the training samples (Figure 5B,E). Increasing the degree of non-linearity in the QSRR model itself and the method formulation should lead to further improvements, especially in the second case study involving peptides > 5 kDa for which the relationship between molecular descriptors and retention time is non-linear [11,12].

Finally, all the analytes predicted using the NLP-based QSRR elution order prediction method fall within their respective chemical domains of applicability. This is evident from Figure 5C,F whereby for both columns the points are within the warning limits of three multiples of standard deviations of standardized residuals and critical leverage values. The QSRR models are thereby considered stable and robust for small organic molecules and peptides up to 24 peptides (the longest peptide in the dataset).

## 3. Methodology

### 3.1. Chromatographic Experiments

Chromatographic experiments performed to obtain the data for development of the NLP-based elution order prediction method are detailed in refs. [13,14]. Briefly, for both case studies gradient elution was used. For the first case study the mobile phase was comprised of methanol and 100 mM tris buffer at pH values of 2.5 and 7.2, while for the second case study it was water with 0.12% trifluoroacetic acid (TFA), and acetonitrile with 0.10% TFA. The 62 organic analytes were dissolved in the mixture of methanol and tris buffer, whereas the 98 synthetic peptides were dissolved in water containing 0.10% of TFA. Dead volumes were determined based on the elution of the second solvents. All the measurements were performed with a flow rate of 1 mL/min, and the injected volume of 20 µL. UV detection was used in both case studies, with wavelengths of 214 and 223 nm, for the first and second case study, respectively. In the first case study, the Supelcosil LC-18 column was used, whereas for the second case study: Xterra MS C18, LiChrospher RP-18, LiChrospher CN, Discovery HS F5-3, Discovery RP Amide C16, PLRP-S and Chromolith columns were used.

### 3.2. QSRR Model Development

Upon obtaining the experimental retention data from literature [13,14], two QSRR models were developed. The QSRR model formulation for the first case study was a simple model involving three parameters defined with the following relationship:

(1) $$t_{R}=f\left(\mu ,\delta_{min},SASA\right)$$

where *µ* is the total dipole moment, *δ_min_* is the natural bond orbital (NBO) [15,16] charge of the most negatively charged atom, while SASA is the solvent accessible surface area. The molecular descriptors for QSRR model defined with Equation (1) were originally obtained using a low-level of theory (MM/AM1) [13]. So, in this work, we have re-optimized the molecular structures and computed all the descriptors using high-level density functional theory (DFT) [17] calculations using the Minnesota 15 (MN15) functional [18] and the 6-311+G ** basis set. [19] The solvation model density (SMD) solvation model [20] with water as a solvent was used to model the pronounced solvent effects. The DFT calculations were performed in Gaussian 16 software (Ref. S1).

In the second case study, a QSRR formulation specifically devised for RP-LC separation of peptides was used:

(2) $$t_{R}=f\left(\log Sum_{AA},\log vdW_{vol.},c\log P\right)$$

where log*Sum*_AA_ is the logarithm of the sum of gradient retention times of 20 natural amino acids, log*vdW*_vol._ is the logarithm of van der Waals volume, and *c*log*P* is the in silico octanol-water partition coefficient describing hydrophobicity.

Commonly, the functional forms of Equations (1) and (2) are linear with coefficients estimated using the MLR method [10].

### 3.3. QSRR Model Validation

#### 3.3.1. External Validation

Both datasets were uniformly separated into training and external validation sets (70/30%) using the Kennard and Stone algorithm [21]. Such external validation was used for the MLR (control), and the MLR-NLP QSRR models. Performance metrics such as the percentage root mean square error (%RMSE) were evaluated and predictive ability of the developed models was also depicted. %RMSE [22,23] was defined as:

(3) $$\%RMSE=\sqrt{\frac{\sum_{i=1}^{n}{\left(\frac{{\hat{y}}_{i}-y_{i}}{y_{i}}\right)}^{2}}{n}}\times 100$$

where *i* is the *i*-th out of *n* compounds, while y_i_ and ŷ_i_ are experimental and predicted retention times, respectively. After predicting the retention times and sorting them w.r.t. the experimental ones, computing the predicted elution order is straight-forward. For %RMSE of elution order, the retention time parameter is simply replaced with the analyte index.

#### 3.3.2. Applicability Domain

Chemical applicability of the QSRR models to a large set of compounds is one of the approaches of their validation. The concept of applicability domain (AD) is introduced for that purpose. AD represents the domain in which compounds possess similar structural, physicochemical or biological properties to the ones of the training compounds. Typical graphical description of the AD is the dependence between standardized residuals of the model and the corresponding leverage values (Williams plot). Leverage values are calculated as the diagonal of the Hat matrix:

(4) $$h=diag\left[X_{2}{}^{T}{\left(X_{2}{}^{T}X_{1}\right)}^{-1}X_{2}\right]$$

where X_1_ is the training set matrix of descriptors, whereas X_2_ can correspond to both training and validation set matrix of descriptors.

To determine whether a compound falls within the AD; warning limits: the critical leverage value h* and three multiples of standard deviation of standardized residuals are determined. The critical leverage value is defined as [22,24]:

(5) $$h^{*}=\frac{3\left(K-1\right)}{N}$$

where *N* is the number of observations, and *K* is the number of variables.

### 3.4. Elution Order Prediction

In this work, an NLP formulation for elution order prediction with relaxed inequality constraints was defined. For a QSRR model with three descriptors $\left[x_{j,1},x_{j,2},x_{j,3}\right]$ for a compound *j* and the corresponding retention time $y_{j}$ sorted in ascending order $\left(y_{j}\leq y_{j+1}\right)$, the QSRR in the optimization formulation can be defined as:

(6) $$\underset{a}{min}\underset{j}{\sum }{\left(y_{j}-{\hat{y}}_{j}\right)}^{2}$$

where $y_{j}=f\left(x_{1},x_{2},x_{3}\right)$ and $f\left(x_{1},x_{2},x_{3}\right)$ can have any functional form. Thereby, Equation (6) becomes:

(7) $$\underset{a}{min}\underset{j}{\sum }{\left(y_{j}-{\hat{y}}_{j}\right)}^{2}=\underset{a}{min}\underset{j}{\sum }{\left(y_{j}-a_{1}x_{j,1}-a_{2}x_{j,2}-a_{3}x_{j,3}\right)}^{2}$$

when *x_j,i_* and *y*_j_ are mean-centered and MLR is used.

This formulation is thereby an NLP problem. When the retention times are sorted in ascending order it is straight-forward to calculate the predicted elution order. From the point of view of mathematical programming, this problem can be handled by adding inequality constraints:

(8) $$\begin{array}{l} \underset{a}{min}\underset{j}{\sum }{\left(y_{j}-a_{1}x_{j,1}-a_{2}x_{j,2}-a_{3}x_{j,3}\right)}^{2}\\ s.t.\\ y_{j}\leq y_{j+1}\;\mathrm{or}\\ a_{1}x_{j,1}-a_{2}x_{j,2}-a_{3}x_{j,3}\leq a_{1}x_{j+1,1}-a_{2}x_{j+1,2}-a_{3}x_{j+1,3} \end{array}$$

However, the resulting constrained NLP problem comprises of too severe inequality constraints which cannot be satisfied and at the same time provide a meaningful QSRR model even for simple mixtures which are the case studies in this thesis.

This was solved by employing relaxed inequality constraints, after which the problem defined by Equation (8) becomes:

(9) $$\begin{array}{l} \underset{\overset{-}{a}}{min}\left\{\overset{m}{\underset{j=1}{\sum }}{\left(y_{j}-a_{1}x_{j,1}-a_{2}x_{j,2}-a_{3}x_{j,3}\right)}^{2}+\overset{m}{\underset{j=1}{\sum }}\alpha_{j}\right\}\\ s.t.\\ a_{1}\left(x_{j,1}-x_{j+1,1}\right)-a_{2}\left(x_{j,2}-x_{j+1,2}\right)-a_{3}\left(x_{j,3}-x_{j+1,3}\right)-\alpha_{j}\leq 0 \end{array}$$

where *α*_j_ is a positive relaxation parameter, whereas ā is a vector of decision variables consisting of *a*_1_, *a*_2_, *a*_3_, and *α*_j_ (*j* = 1, 2, …, *m* − 1). For solving this NLP formulation for chromatographic elution order prediction, in this work, the interior-point algorithm [25,26] has been used.

## 4. Conclusions

In conclusion, an NLP-based elution order prediction method has been developed and tested on two case studies involving simple analytical mixtures. In all the case studies, across all the columns and all the chromatographic conditions, the percentage root mean square error (%RMSE) of retention time increased for 29.13%, while the %RMSE of elution order decreased by 37.29%.

Therefore, sacrificing %RMSE(*t*_R_) led to a considerable increase in the elution order predictive ability of the QSRR models when compared to the control MLR models. As compared to the previous study employing multi-objective optimization, the presented method is considerably faster, making it suitable for implementation in commercial chromatographic environment and LC-MS/MS workflows. Our future work will envelop the large-scale application of the derived NLP-based formulation of elution order prediction to complex mixtures such as proteomics and metabolomics where it can facilitate peptide/metabolite identification.

## Figures and Tables

**Table 1:** Summary of the paired *t*-test for all the QSRR model performances for all the columns between the two approaches (MLR and MLR-NLP).

Statistics	%RMSE(t_R_) MLR	%RMSE(t_R_) MLR-NLP
Mean	26.635	36.848
Variance	135.67	490.97
Observations	19	19
Pearson Correlation	0.961	
Df	18	
t Stat	−3.897	
P(T<=t) one-tail	0.00053	
t Critical one-tail	1.734	
P(T<=t) two-tail	0.00106	
t Critical two-tail	2.100	

**Table 2:** Summary of model performances for the first and second case studies.

CS ^a^	Column	Analysis Parameters ^b^	Model	%RMSE(*t*_R_)	%RMSE(order)
I	Supelcosil	*t*_G_ = 10 min, *T* = 35 °C	MLR (control)	8.57	59.07
			MLR-NLP	8.07	51.77
II	Xterra	*t*_G_ = 20 min, *T* = 40 °C	MLR (control)	11.50	25.01
			MLR-NLP	15.17	22.40
II	Licrospher	*t*_G_ = 20 min, *T* = 40 °C	MLR (control)	13.25	30.28
			MLR-NLP	12.42	39.59
II	Licrospher	*t*_G_ = 60 min, *T* = 40 °C	MLR (control)	25.60	34.11
			MLR-NLP	37.94	30.10
II	Licrospher	*t*_G_ = 120 min, *T* = 40 °C	MLR (control)	42.31	153.00
			MLR-NLP	85.62	25.17
II	Licrospher	*t*_G_ = 20 min, *T* = 60 °C	MLR (control)	18.45	36.12
			MLR-NLP	16.86	40.70
II	Licrospher	*t*_G_ = 20 min, *T* = 80 °C	MLR (control)	18.82	35.25
			MLR-NLP	21.06	34.65
II	Licrospher	*t*_G_ = 20 min, *T* = 40 °C	MLR (control)	39.28	195.82
			MLR-NLP	55.53	53.45
II	PRP	*t*_G_ = 20 min, *T* = 40 °C	MLR (control)	20.07	69.44
			MLR-NLP	20.72	58.09
II	PRP	*t*_G_ = 60 min, *T* = 40 °C	MLR (control)	37.92	107.94
			MLR-NLP	52.40	41.33
II	PRP	*t*_G_ = 20 min, *T* = 60 °C	MLR (control)	21.75	94.97
			MLR-NLP	24.06	82.54
II	PRP	*t*_G_ = 60 min, *T* = 60 °C	MLR (control)	40.11	321.65
			MLR-NLP	54.35	37.16
II	PRP	*t*_G_ = 20 min, *T* = 80 °C	MLR (control)	22.36	137.16
			MLR-NLP	26.19	53.30
II	PRP	*t*_G_ = 60 min, *T* = 80 °C	MLR (control)	42.60	194.56
			MLR-NLP	61.56	40.18
II	Discovery	*t*_G_ = 20 min, *T* = 40 °C	MLR (control)	36.73	261.22
			MLR-NLP	58.07	91.81
II	Discovery	*t*_G_ = 20 min, *T* = 60 °C	MLR (control)	36.37	219.01
			MLR-NLP	57.16	96.70
II	Discovery	*t*_G_ = 20 min, *T* = 80 °C	MLR (control)	36.74	241.63
			MLR-NLP	54.75	81.05
II	Discovery	*t*_G_ = 20 min, *T* = 40 °C	MLR (control)	12.81	34.00
			MLR-NLP	13.84	28.12
II	Chromolith	*t*_G_ = 20 min, *T* = 40 °C	MLR (control)	20.82	43.81
			MLR-NLP	24.36	28.55

^a^ CS—case study; ^b^ *t*_G_—gradient retention time; MLR—multiple linear regression; MLR-NLP—multiple linear regression–non-linear programming.

## Supplementary Materials

Supplementary materials can be found at https://www.mdpi.com/1422-0067/20/14/3443/s1.
