# Supplementary material for: Quantitative Structure–Retention Relationships with Non-Linear Programming for Prediction of Chromatographic Elution Order
Source: Int J Mol Sci. 2019 Jul 12;20(14):3443. doi: 10.3390/ijms20143443 (PMC6678770; doi:10.3390/ijms20143443)
Supplement: Supplementary file 1 [file ijms-20-03443-s001.pdf]

Article

Quantitative structure-retention relationships with non-linear programming for prediction of chromatographic elution order

J. Jay Liu[1], Alham Alipuly[1], Tomasz Bączek[2], Ming Wah Wong[3] and Petar Žuvela[3,*]

[1] Department of Chemical Engineering, Pukyong National University, 48-513, Busan, Korea; jayliu@pknu.ac.kr (J.J.L.); alipuy@pukyong.ac.kr (A.A.)
[2] Department of Pharmaceutical Chemistry, Medical University of Gdańsk, Al. Gen. Hallera 107, 80-416, Gdańsk, Poland; tbaczek@gumed.edu.pl (T.B.)
[3] Department of Chemistry, National University of Singapore, 3 Science Drive 3, Singapore 117543, Singapore; chmwmw@nus.edu.sg (M.W.W.)
* Correspondence: petar.zuvela@nus.edu.sg (P. Ž.)

Supporting Information

Contents:

Reference S1. Complete Gaussian 16 reference.

Figures S1-S10. Performance plots for MLR-NLP elution order prediction method for all the chromatographic columns and both case studies.

Ref. S1. Gaussian 16, Revision B.01, Frisch, M. J.; Trucks, G. W.; Schlegel, H. B.; Scuseria, G. E.; Robb, M. A.; Cheeseman, J. R.; Scalmani, G.; Barone, V.; Petersson, G. A.; Nakatsuji, H.; Li, X.; Caricato, M.; Marenich, A. V.; Bloino, J.; Janesko, B. G.; Gomperts, R.; Mennucci, B.; Hratchian, H. P.; Ortiz, J. V.; Izmaylov, A. F.; Sonnenberg, J. L.; Williams-Young, D.; Ding, F.; Lipparini, F.; Egidi, F.; Goings, J.; Peng, B.; Petrone, A.; Henderson, T.; Ranasinghe, D.; Zakrzewski, V. G.; Gao, J.; Rega, N.; Zheng, G.; Liang, W.; Hada, M.; Ehara, M.; Toyota, K.; Fukuda, R.; Hasegawa, J.; Ishida, M.; Nakajima, T.; Honda, Y.; Kitao, O.; Nakai, H.; Vreven, T.; Throssell, K.; Montgomery, J. A., Jr.; Peralta, J. E.; Ogliaro, F.; Bearpark, M. J.; Heyd, J. J.; Brothers, E. N.; Kudin, K. N.; Staroverov, V. N.; Keith, T. A.; Kobayashi, R.; Normand, J.; Raghavachari, K.; Rendell, A. P.; Burant, J. C.; Iyengar, S. S.; Tomasi, J.; Cossi, M.; Millam, J. M.; Klene, M.; Adamo, C.; Cammi, R.; Ochterski, J. W.; Martin, R. L.; Morokuma, K.; Farkas, O.; Foresman, J. B.; Fox, D. J. Gaussian, Inc., Wallingford CT, 2016.

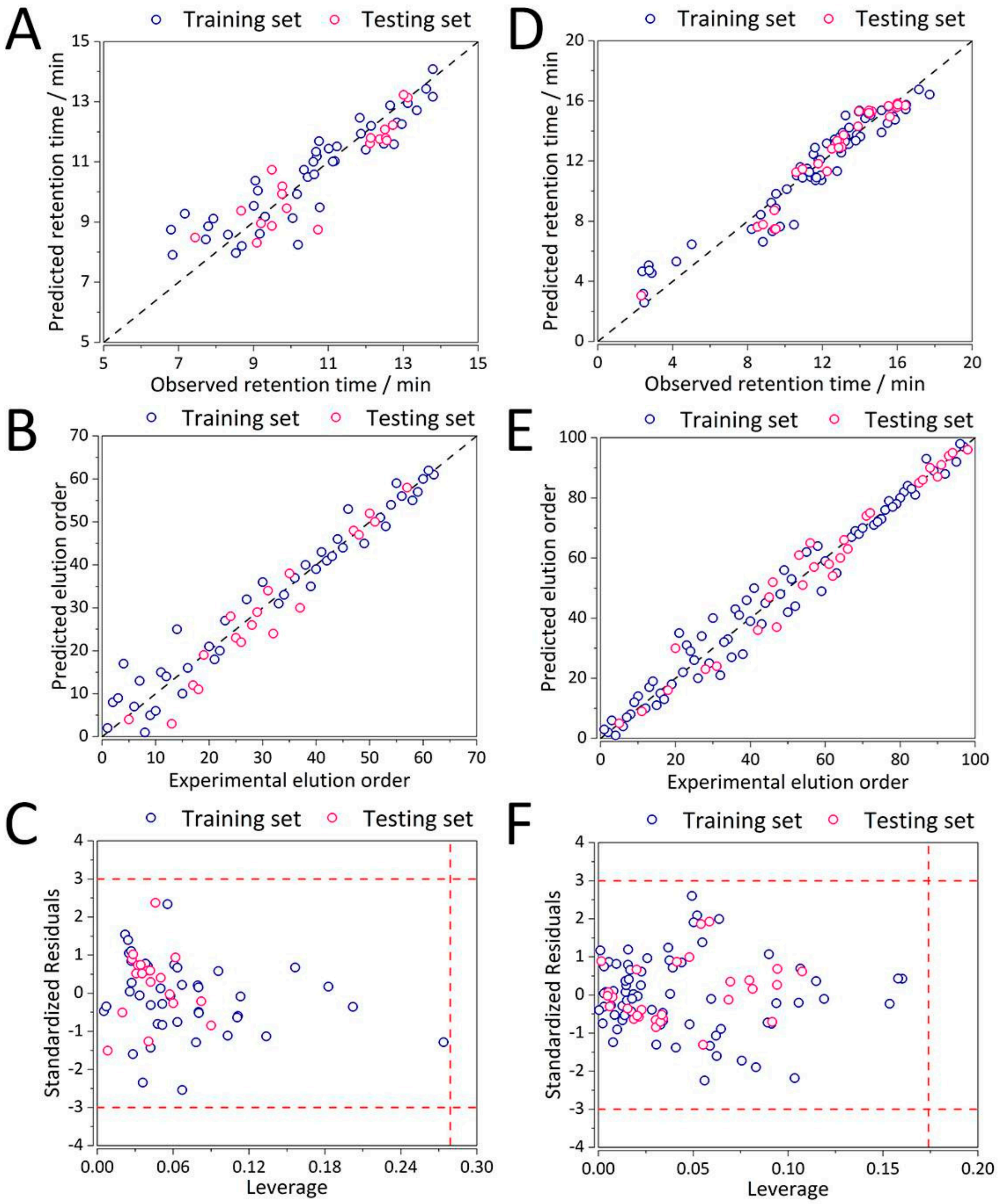

Figure S1. Performance of the MLR-NLP method for prediction of **A)** retention time, **B)** elution order, and **C)** applicability domain for case study 1 (separation of organic molecules using Supelcosil LC, t_G = 10 min, T = 35 °C), **D)** prediction of retention time, **E)** elution order, and **F)** applicability domain for case study 2 (separation of synthetic peptides on Xterra, t_G = 20 min, T = 40 °C).

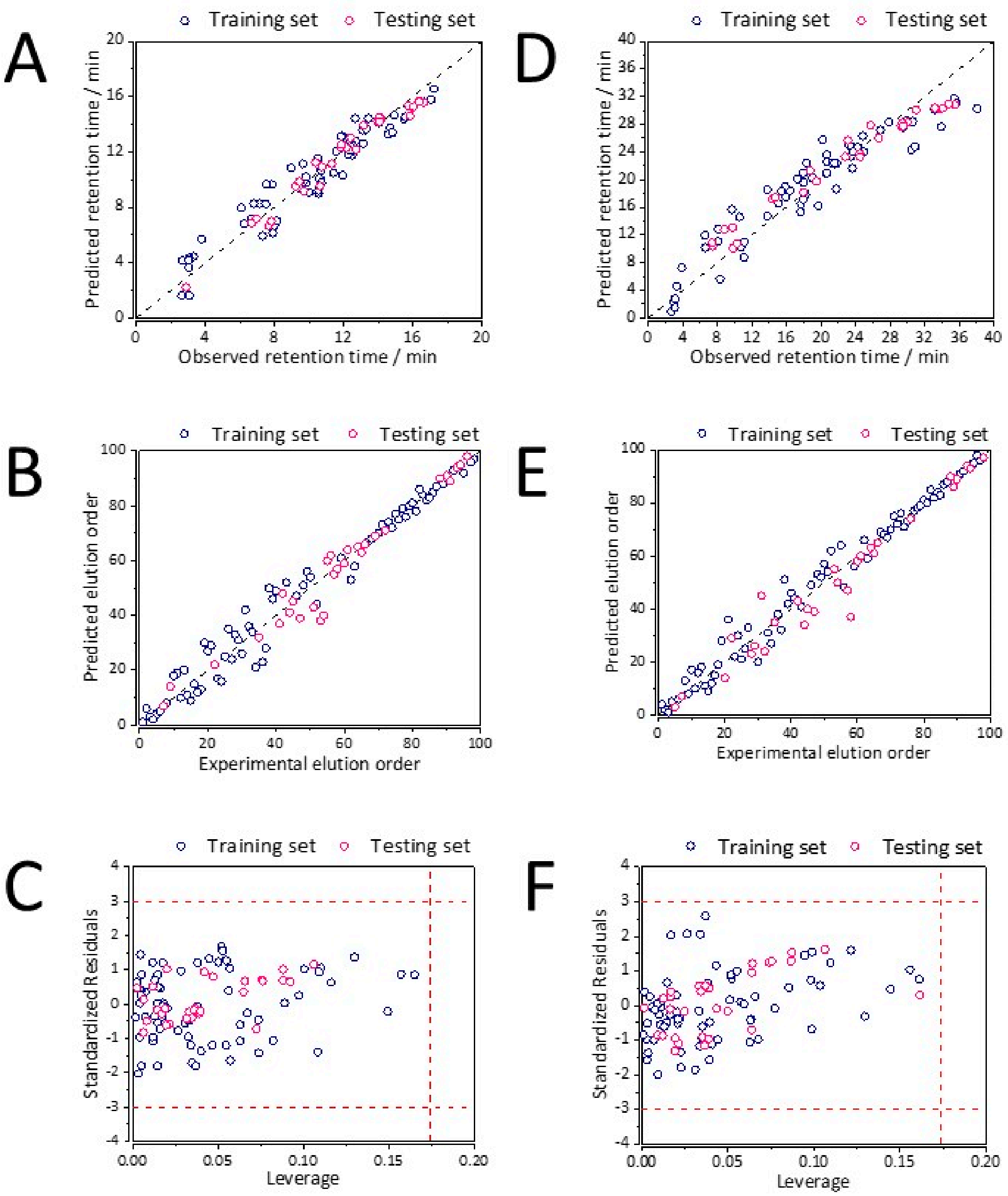

Figure S2. Performance of the MLR-NLP method for prediction of **A)** retention time, **B)** elution order, and **C)** applicability domain for case study 2 (separation of synthetic peptides on Licrospher, $t_G = 20$ min, $T = 40$ °C). **D)** prediction of retention time, **E)** elution order, and **F)** applicability domain for case study 2 (separation of synthetic peptides on Licrospher, $t_G = 60$ min, $T = 40$ °C).

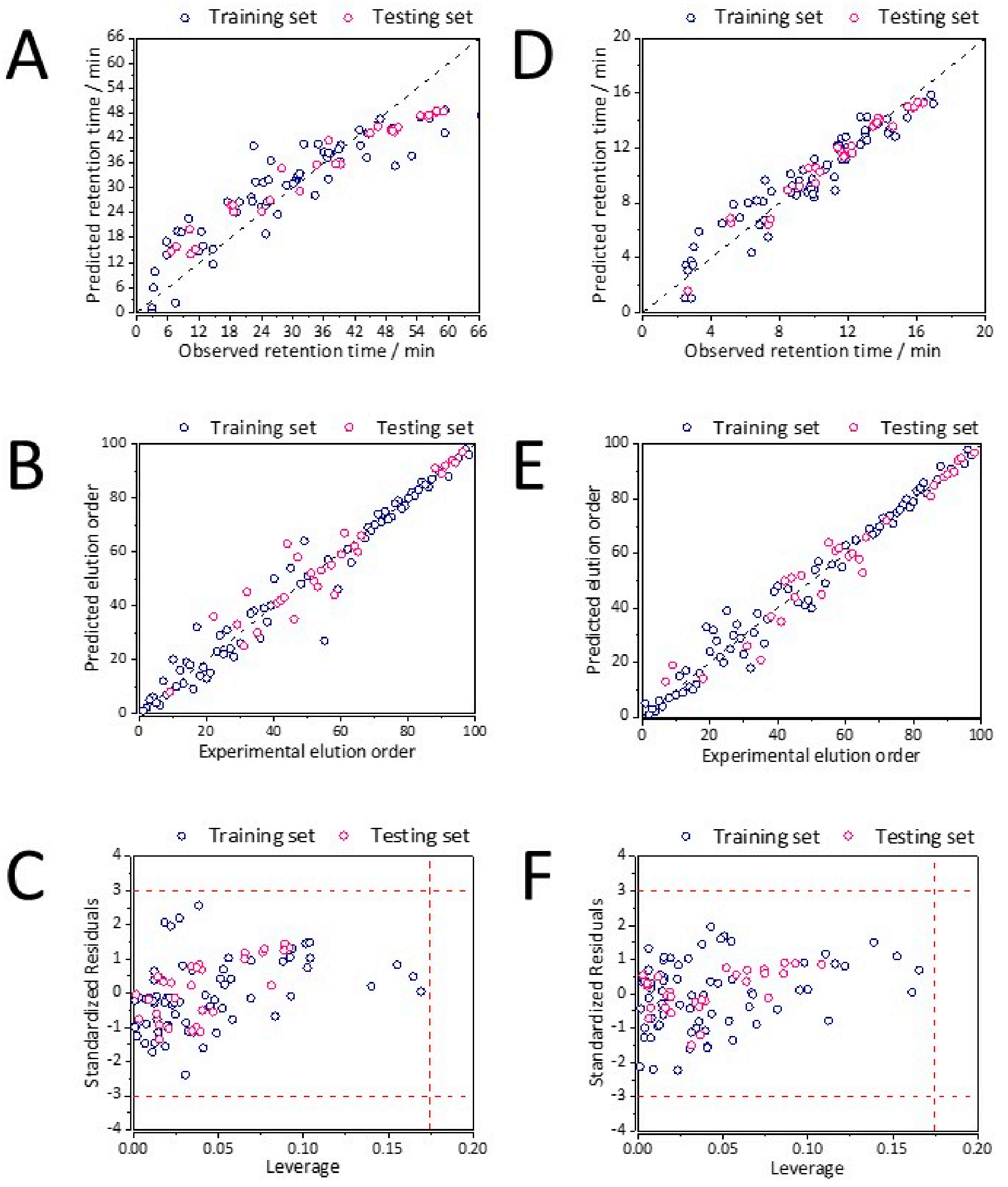

Figure S3. Performance of the MLR-NLP method for prediction of **A)** retention time, **B)** elution order, and **C)** applicability domain for case study 2 (separation of synthetic peptides on Licrospher, $t_G = 120$ min, $T = 40$ °C). **D)** prediction of retention time, **E)** elution order, and **F)** applicability domain for case study 2 (separation of synthetic peptides on Licrospher, $t_G = 20$ min, $T = 60$ °C).

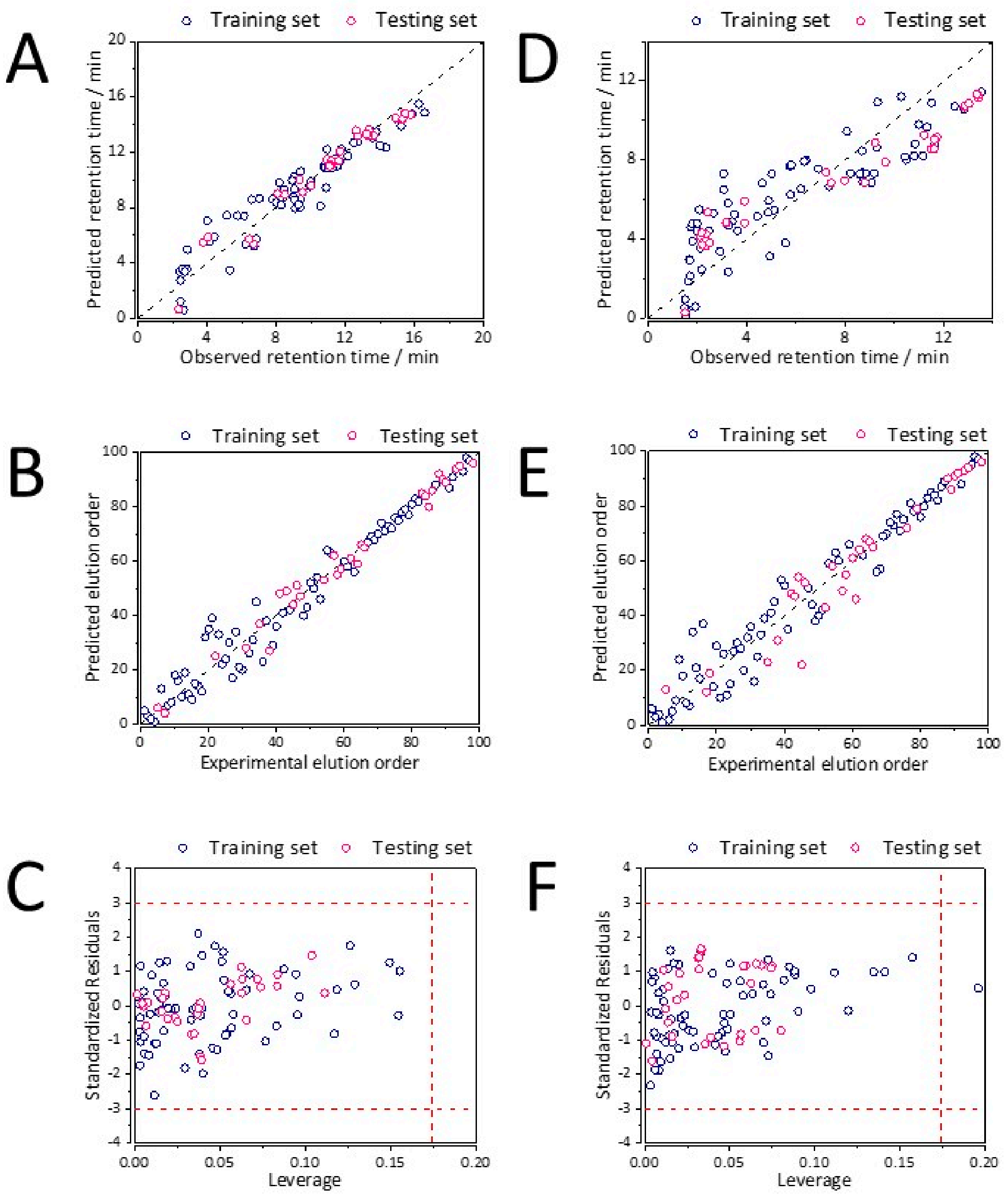

Figure S4. Performance of the MLR-NLP method for prediction of **A)** retention time, **B)** elution order, and **C)** applicability domain for case study 2 (separation of synthetic peptides on Licrospher, t_G = 20 min, T = 80 °C). **D)** prediction of retention time, **E)** elution order, and **F)** applicability domain for case study 2 (separation of synthetic peptides on Licrospher, t_G = 20 min, T = 40 °C).

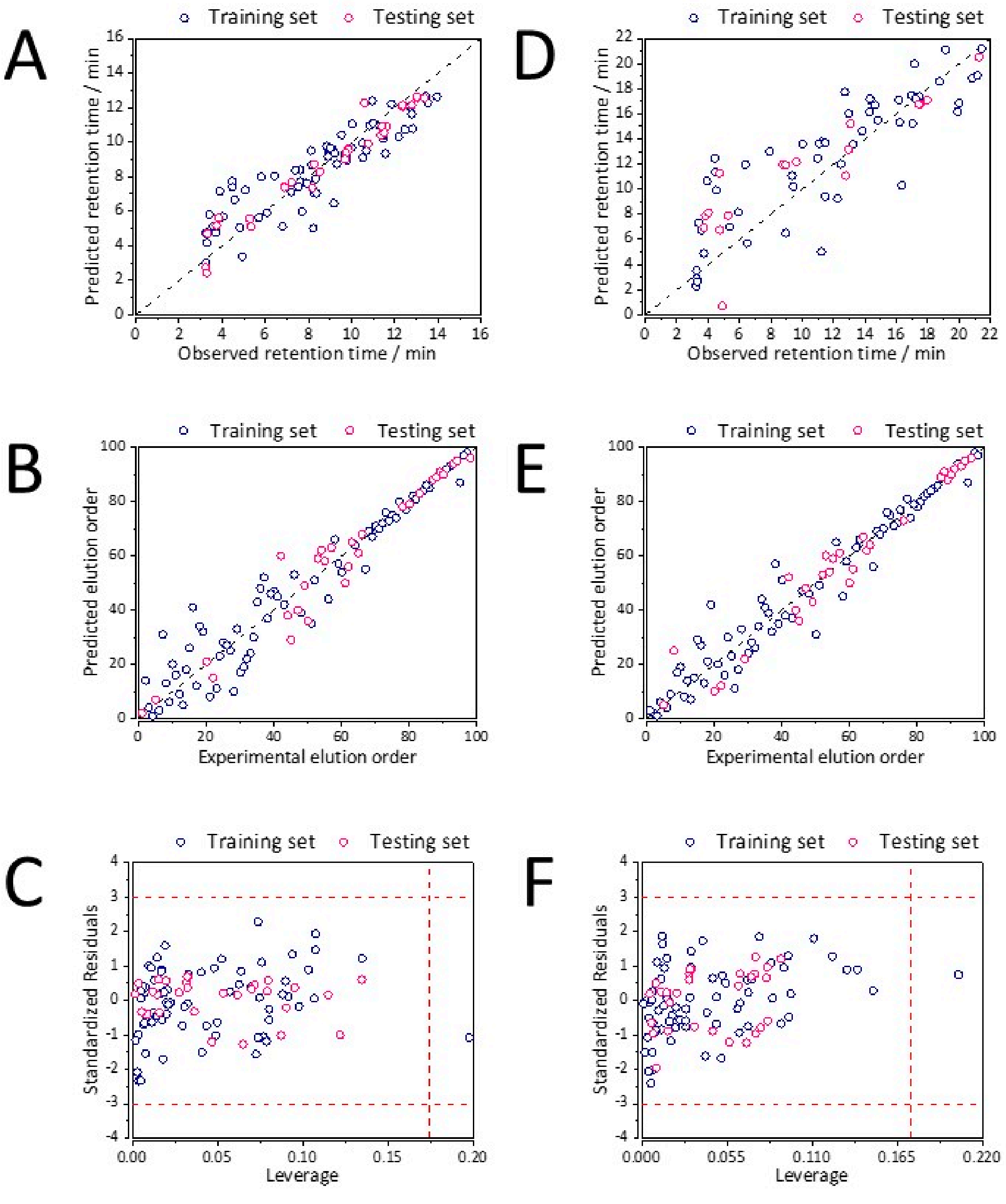

Figure S5. Performance of the MLR-NLP method for prediction of **A)** retention time, **B)** elution order, and **C)** applicability domain for case study 2 (separation of synthetic peptides on PRP, t_G = 20 min, T = 40 °C). **D)** prediction of retention time, **E)** elution order, and **F)** applicability domain for case study 2 (separation of synthetic peptides on PRP, t_G = 60 min, T = 40 °C).

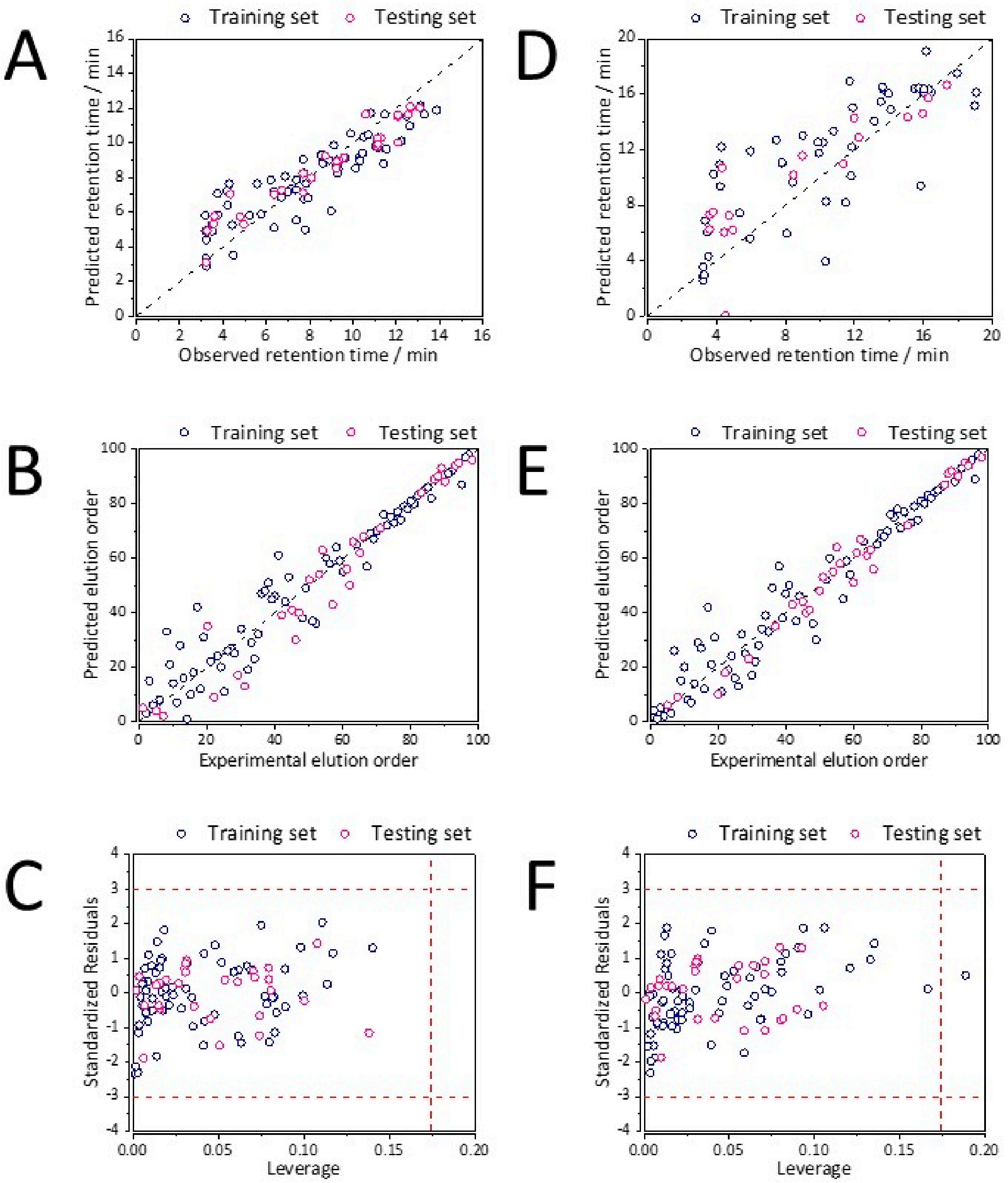

Figure S6. Performance of the MLR-NLP method for prediction of **A)** retention time, **B)** elution order, and **C)** applicability domain for case study 2 (separation of synthetic peptides on PRP, t_G = 20 min, T = 60 °C). **D)** prediction of retention time, **E)** elution order, and **F)** applicability domain for case study 2 (separation of synthetic peptides on PRP, t_G = 60 min, T = 60 °C).

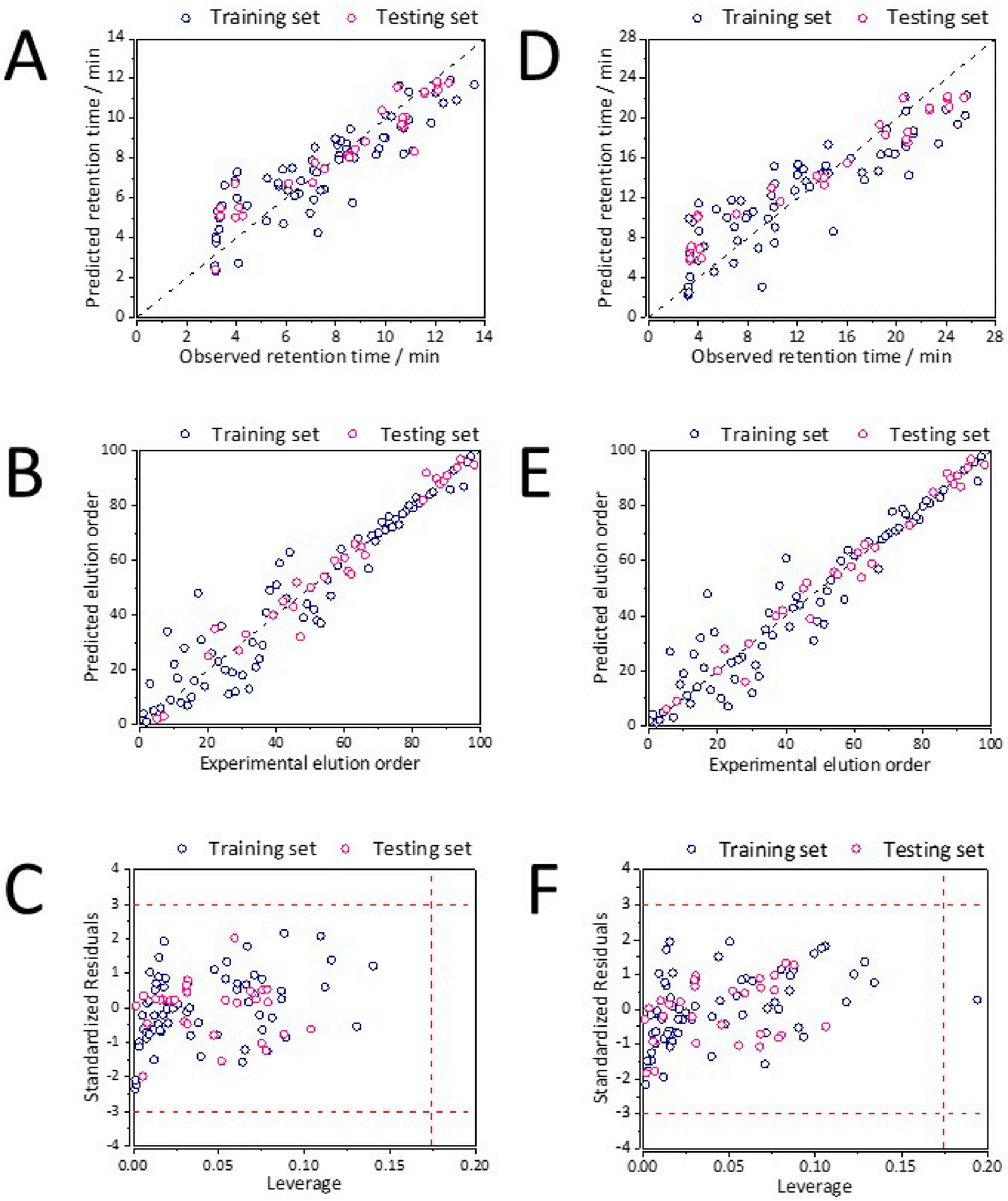

Figure S7. Performance of the MLR-NLP method for prediction of **A)** retention time, **B)** elution order, and **C)** applicability domain for case study 2 (separation of synthetic peptides on PRP, t_G = 20 min, T = 80 °C). **D)** prediction of retention time, **E)** elution order, and **F)** applicability domain for case study 2 (separation of synthetic peptides on PRP, t_G = 60 min, T = 80 °C).

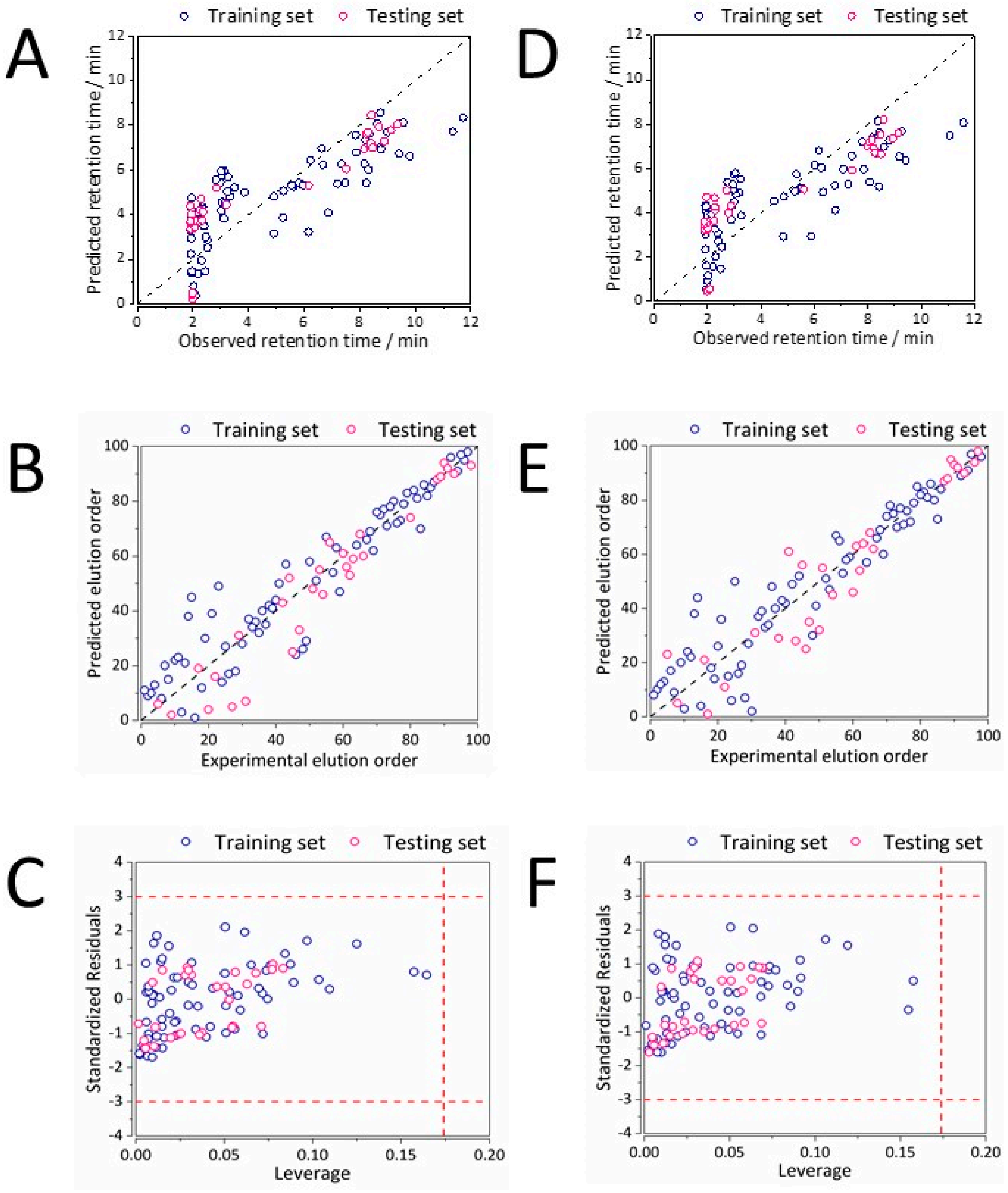

Figure S8. Performance of the MLR-NLP method for prediction of **A)** retention time, **B)** elution order, and **C)** applicability domain for case study 2 (separation of synthetic peptides on Discovery, t_G = 20 min, T = 40 °C). **D)** prediction of retention time, **E)** elution order, and **F)** applicability domain for case study 2 (separation of synthetic peptides on Discovery, t_G = 20 min, T = 60 °C).

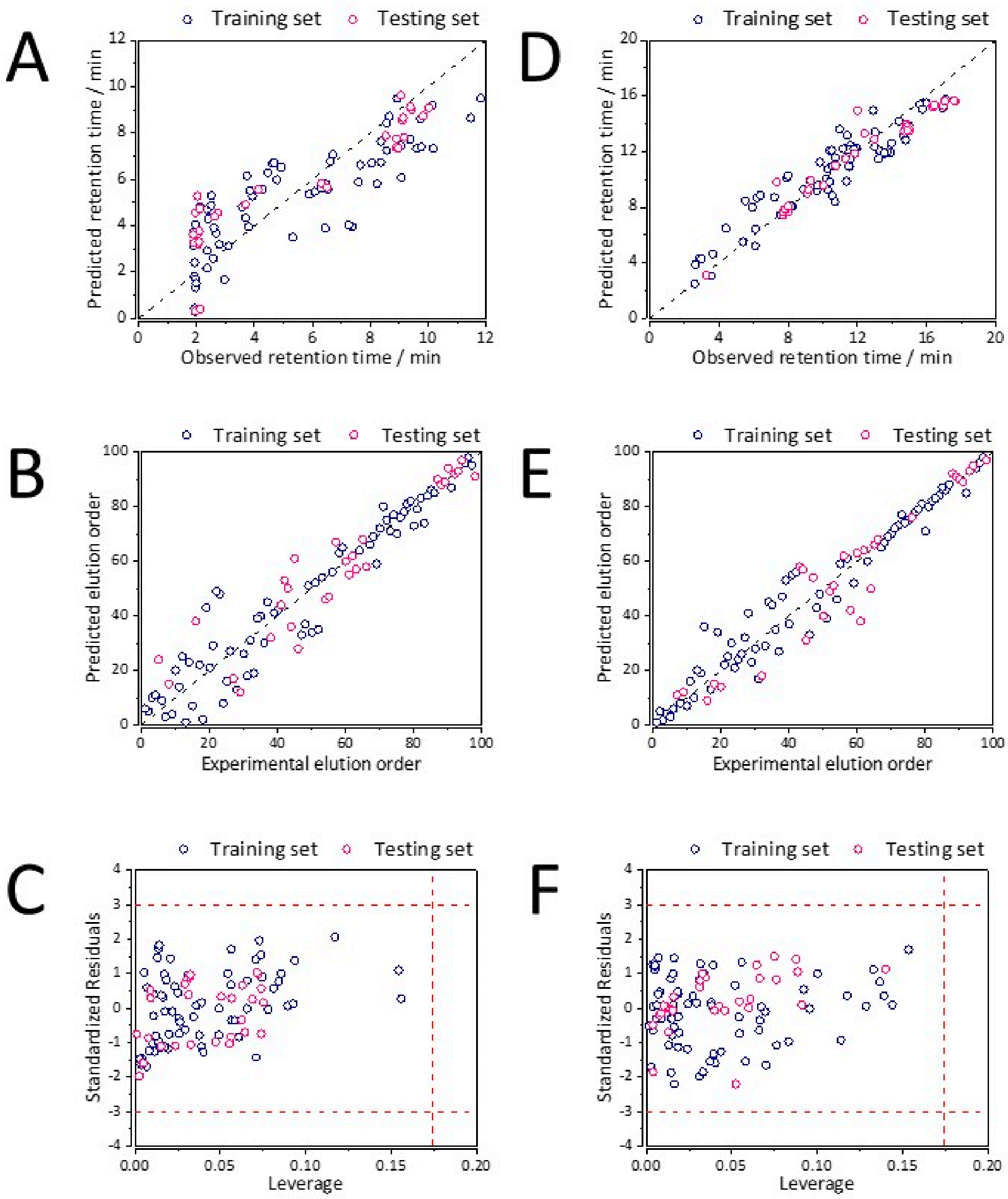

Figure S9. Performance of the MLR-NLP method for prediction of **A)** retention time, **B)** elution order, and **C)** applicability domain for case study 2 (separation of synthetic peptides on Discovery, t_G = 20 min, T = 80 °C). **D)** prediction of retention time, **E)** elution order, and **F)** applicability domain for case study 2 (separation of synthetic peptides on Discovery, t_G = 20 min, T = 40 °C).

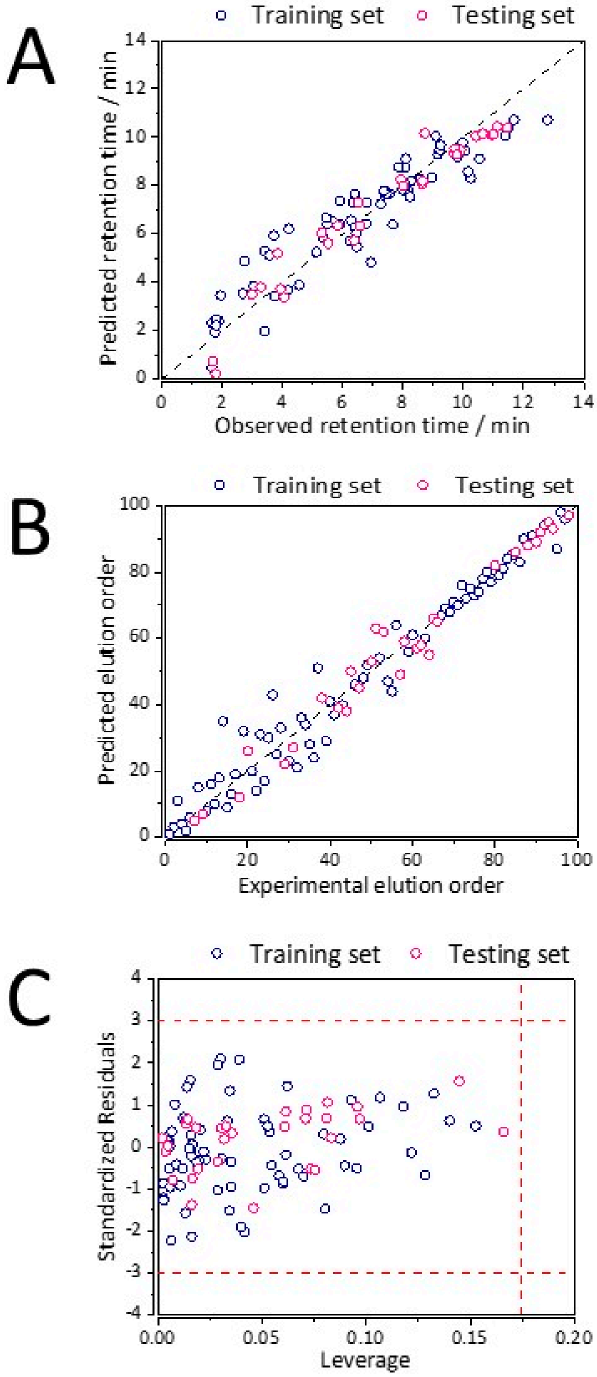

Figure S10. Performance of the MLR-NLP method for prediction of **A)** retention time, **B)** elution order, and **C)** applicability domain for case study 2 (separation of synthetic peptides on Chromolith, t_G = 20 min, T = 40 °C).
